# Multi-Omics Analysis Reveals the Distinct Features of Metabolism Pathways Supporting the Fruit Size and Color Variation of Giant Pumpkin

**DOI:** 10.3390/ijms25073864

**Published:** 2024-03-29

**Authors:** Wenhao Xia, Chen Chen, Siying Jin, Huimin Chang, Xianjun Ding, Qinyi Fan, Zhiping Zhang, Bing Hua, Minmin Miao, Jiexia Liu

**Affiliations:** 1 College of Horticulture and Landscape Architecture, Yangzhou University, Yangzhou 225009, China221803104@stu.yzu.edu.cn (S.J.); 221803101@stu.yzu.edu.cn (H.C.); 221803103@stu.yzu.edu.cn (Q.F.); binghua@yzu.edu.cn (B.H.); mmmiao@yzu.edu.cn (M.M.); 2 Joint International Research Laboratory of Agriculture and Agri-Product Safety of Ministry of Education of China, Yangzhou University, Yangzhou 225009, China; 3 Key Laboratory of Plant Functional Genomics of the Ministry of Education, Yangzhou University, Yangzhou 225009, China

**Keywords:** giant pumpkin, fruit size, fruit color, transcriptome, proteome, metabolome

## Abstract

Pumpkin (*Cucurbita maxima*) is an important vegetable crop of the Cucurbitaceae plant family. The fruits of pumpkin are often used as directly edible food or raw material for a number of processed foods. In nature, mature pumpkin fruits differ in size, shape, and color. The Atlantic Giant (AG) cultivar has the world’s largest fruits and is described as the giant pumpkin. AG is well-known for its large and bright-colored fruits with high ornamental and economic value. At present, there are insufficient studies that have focused on the formation factors of the AG cultivar. To address these knowledge gaps, we performed comparative transcriptome, proteome, and metabolome analysis of fruits from the AG cultivar and a pumpkin with relatively small fruit (Hubbard). The results indicate that up-regulation of gene-encoded expansins contributed to fruit cell expansion, and the increased presence of photoassimilates (stachyose and D-glucose) and jasmonic acid (JA) accumulation worked together in terms of the formation of large fruit in the AG cultivar. Notably, perhaps due to the rapid transport of photoassimilates, abundant stachyose that was not converted into glucose in time was detected in giant pumpkin fruits, implying that a unique mode of assimilate unloading is in existence in the AG cultivar. The potential molecular regulatory network of photoassimilate metabolism closely related to pumpkin fruit expansion was also investigated, finding that three MYB transcription factors, namely CmaCh02G015900, CmaCh01G018100, and CmaCh06G011110, may be involved in metabolic regulation. In addition, neoxanthin (a type of carotenoid) exhibited decreased accumulation that was attributed to the down-regulation of carotenoid biosynthesis genes in AG fruits, which may lead to pigmentation differences between the two pumpkin cultivars. Our current work will provide new insights into the potential formation factors of giant pumpkins for further systematic elucidation.

## 1. Introduction

Horticultural crops possess a wide variety of fruits. Fruits produced from different cultivars of the same species vary in size, color, shape, and flavor, and some cultivars have been targeted for domestication and genetic engineering modification due to their nutritional/ornamental value and delicious taste [1,2,3]. The formation and development of fruits is regulated by multifaceted factors [4,5]. Fruit expansion is usually achieved through an increase in the number of fruit cells, fruit cell expansion, and the accumulation of photoassimilates [6]. A previous study investigated the relationship between cell size and sucrose accumulation in cucurbit fruits under mechanical restriction and found that restricting fruit through the use of stainless steel globular cages resulted in a larger number of smaller cells and decreased sucrose accumulation in the fruit [7]. 

The Cucurbitaceae family includes a lot of economically important fruits, such as cucumber, pumpkin, watermelon, melon, and bitter melon. The Cucurbitaceae fruits are well known due to their diverse size, shape, and color, and there are a number of studies that investigate their genetic basis and molecular regulation [8]. In cucumber, the *CsARC6* mutation led to white fruit color, larger fruit epidermal cells, lower carotenoid content, and smaller fruits [9]. Under low-light stress, genes related to secondary metabolites, carbohydrate and amino acid metabolism, and transcriptional regulation affected the fruit expansion of watermelon [10]. The fruit size/shape candidate genes have been identified, including *CsSUN25-26-27a* and *CsTRM5* in cucumber, *CmOFP1a* in melon, and *ClSUN25-26-27a* in watermelon [8]. Research on the molecular mechanism related to differences in the fruit characteristics of Cucurbitaceae plants is still being undertaken.

Pumpkin, one of the earliest ancient species cultivated by humans, is an annual vine herb of the Cucurbitaceae family that exhibits abundant germplasm diversity, high levels of production in terms of amount, and significant commercial value [11]. Currently, pumpkins are cultivated and consumed around the world. Pumpkin fruits and seeds are rich in carotenoids, vitamins, dietary fiber, minerals, and phenolic compounds and can be eaten directly or after processing [11,12,13]. The diversity of pumpkin fruits is a vital characteristic of different pumpkin cultivars, with contrasts including large and small fruits, round and oval-shaped fruits, and orange, yellow, and cyan fruits. According to the size of the fruit, pumpkins are divided into mini, medium, large, and giant varieties. The giant pumpkin, or Atlantic Giant (AG, *Cucurbita maxima*), is known to be the largest fruit (weights 1226 kg) in the world ([14]; http://www.pumpkinnook.com/giants.htm; accessed on 1 February 2023), which is an important feature of it [6]. The fruits of the AG cultivar can be used for exhibition purposes or as part of a personal collection, with interest in this cultivar often attributed to its attractive appearance and long storage period. 

In recent years, cultivating giant pumpkins with larger fruit has attracted the interest of many growers, including quite a few farmers and companies, many of which aim to sell high or achieve the goal of further breaking the world record. It is reported that the currently cultivated giant pumpkin (AG) originated from the Hubbard squash [15]. Moreover, within only 100 years, the fruit weight of the modern AG cultivar has dramatically increased compared to its ancestor [16]. Thus, revealing the variation mode of giant pumpkin in relation to unique fruits will be a worthwhile topic. However, to date, there are few conceptual models to explain how giant pumpkins achieve such remarkable fruit productivity. 

Integrated multi-omics analysis is a powerful tool to identify the formation factors of important agronomic traits and has been widely applied in various species [17,18,19]. In this study, we conducted transcriptomic, proteomic, and metabolomic analyses of the AG cultivar and a pumpkin with small fruit (Hubbard) to explore the metabolic differences and molecular networks associated with variation in giant pumpkin fruits. The findings will enrich our knowledge about the formation of giant pumpkin and provide a theoretical basis for cultivating pumpkin germplasm resources with excellent fruit characteristics. 

## 2. Results

### 2.1. Phenotype of AG and Hubbard Fruits

**Figure 1:**
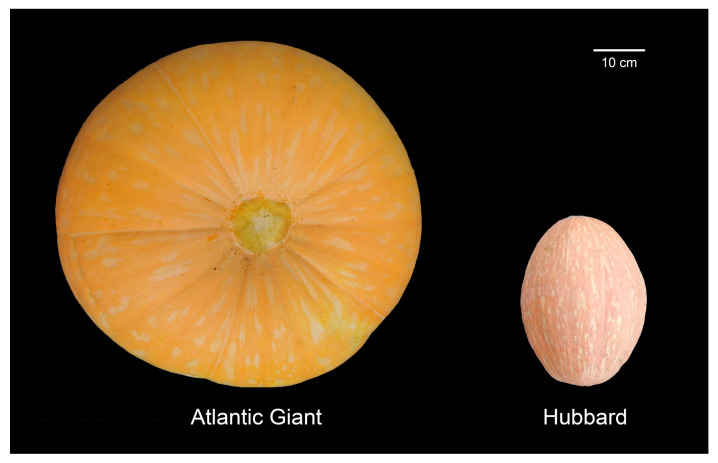
The phenotype of two pumpkin cultivars: Atlantic Giant and Hubbard fruits.

AG and Hubbard fruits were collected at the rapid growth stage (at 30 days after flowering for AG and at 20 days after flowering for Hubbard), with representative fruits shown in Figure 1. Although the fruit weight of the AG plants in this experiment was much lighter than the world’s heaviest fruit record (1226 kg) due to the limited growth condition, we still observed that the fruit of the AG cultivar were much larger than that of the Hubbard cultivar. Additionally, there was an obvious difference in fruit color between these two pumpkin cultivars. The AG cultivar showed orange–yellow-colored fruits, and the Hubbard cultivar showed orange–red-colored fruits.

### 2.2. Transcriptome Sequencing and Analysis

**Figure 2:**
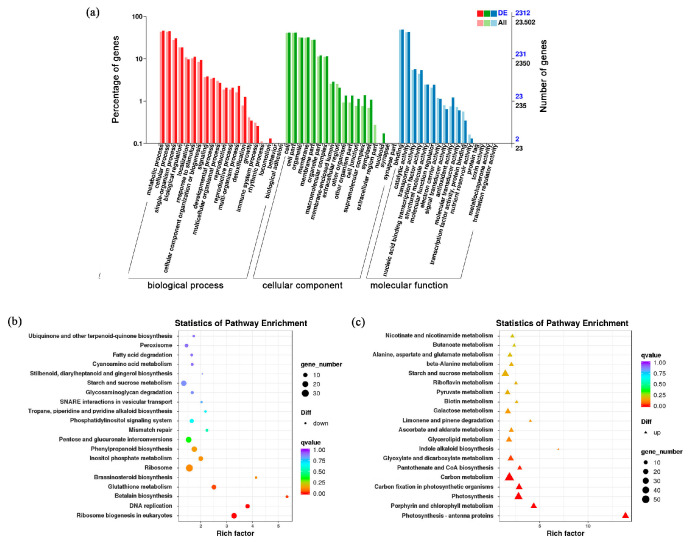
Annotation and pathway analysis of DEGs between AG and Hubbard fruits. (**a**) GO classification of all annotated genes and DEGs. (**b**,**c**) Scatter plot of the most enriched KEGG pathways of down-regulated (**b**) and up-regulated (**c**) DEGs.

PCA (principal component analysis) estimation based on the count matrix of all genes across two genotypes and sample clustering based on the read count matrix were conducted (Figure S1), demonstrating that three independent samples (per pumpkin specie) used for transcriptomic analysis showed great correlation. Considerable differential expression genes (DEGs) were identified from RNA-seq analysis. To further explore the functions of DEGs, GO (Gene Ontology) and KEGG (Kyoto Encyclopedia of Genes and Genomes) pathway annotations were carried out. GO enrichment analysis revealed that the most significantly enriched terms were metabolic process, cell, and binding in the ‘biological process’, ‘cellular component’, and ‘molecular function’ categories, respectively (Figure 2a). The assay of the KEGG enrichment pathway showed that the up-regulated DEGs in AG fruits were significantly enriched in two pathways, namely glutathione metabolism and brassinosteroid biosynthesis, compared with Hubbard fruits. The down-regulated DEGs were closely associated with photosynthesis and carbon metabolism (Figure 2b,c).

### 2.3. Different Expression Patterns of DEGs Associated with Phenotype Variation

**Figure 3:**
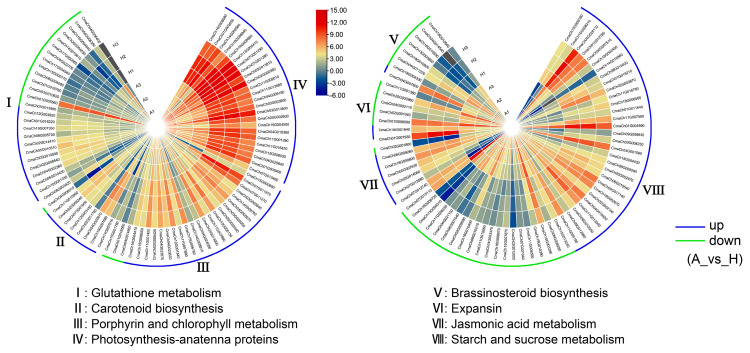
The expression heatmap of DEGs in several KEGG pathways related to phenotypic differentiation between Atlantic Giant and Hubbard fruits. The expression profile was determined based on log_2_(FPKM) in RNA-seq data for each gene. A1~3 and H1~3 represent AG and Hubbard fruits, respectively.

To explain the possible reasons of phenotype differentiation (size and color) between AG and Hubbard fruits, an expression heatmap of DEGs in the significant enriched KEGG pathway and the other pathways of interest (carotenoid biosynthesis, that related to color variation; expansins and jasmonic acid (JA) metabolism, those perhaps related to fruit size variation) was drawn based on RNA-seq data (Figure 3, Table S1). In glutathione metabolism and starch and sucrose metabolism, the number of up-regulated and down-regulated DEGs was similar in a comparison between AG and Hubbard fruits. The majority of DEGs involved in carotenoid biosynthesis, photosynthetic antenna proteins, porphyrin and chlorophyll metabolism, and jasmonic acid metabolism were up-regulated in Hubbard fruits, whereas most of the DEG-encoded expansins (usually linked to cell expansion) and those involved in brassinosteroid biosynthesis showed remarkable up-regulation in AG fruits.

### 2.4. Identification of Expressed Proteins in AG and Hubbard Fruits

**Figure 4:**
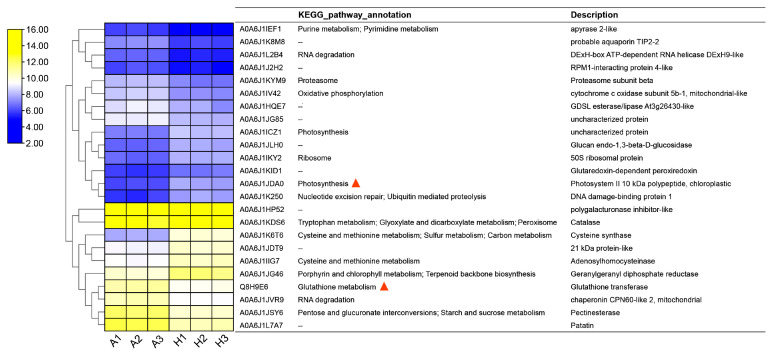
Expression profile and annotation of DEPs identified from a comparison between Atlantic Giant and Hubbard fruits. The expression profile was determined based on log_2_(protein expression) in proteomic data for each protein. Red triangles indicate the protein annotations that we noticed. A1~3 and H1~3 represent AG and Hubbard fruits, respectively.

PCA of the individual sample in proteomics sequencing is shown in Figure S2. In total, 6008 pumpkin proteins were identified, among which, 3985 were function annotated by comparison with the GO, KEGG, and COG databases (Tables S2 and S3; Figure S3). The distribution of annotated proteins in GO classification and KEGG pathways was analyzed (Figure S4). The differentially expressed proteins (DEPs) in AG and Hubbard fruits were found (Figure S5). A total of 24 DEPs between two pumpkin cultivars were discovered (Table S4). The number of identified DEPs was far less than the number of identified DEGs. KEGG pathway analysis of these DEPs suggests that they were mainly involved in the metabolic pathway and photosynthesis (Figure S6). As shown in Figure 4, Q8H9E6 (glutathione transferase) displayed high expression in AG fruits (the expression of corresponding coding genes, i.e., *CmaCh04G006380*, was also up-regulated in AG fruits), indicating that the giant pumpkins might contain higher antioxidant capacity. The A0A6J1JDA0 and A0A6J1ICZ1 proteins concerned with the photosynthesis pathway were up-regulated in Hubbard fruit; this result was consistent with our previously described transcriptome result.

### 2.5. The Fruits of AG and Hubbard Contain Different Metabolite Content

**Figure 5:**
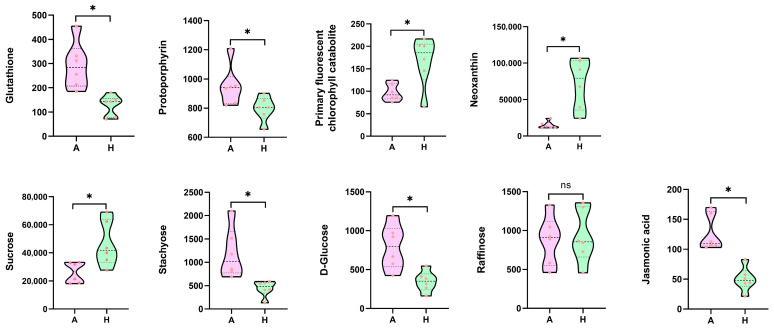
The relative content of metabolites in pumpkin fruits generated from metabolomics data. * represents the differential metabolites between AG and Hubbard fruits. ‘ns’ represents no difference.

To survey the variation in metabolites in AG and Hubbard fruits, the metabolome focusing on metabolites was analyzed using LC-QTOF-MS. Large number of metabolites were identified and quantified. Combined with previously described phenotypes, transcriptomes, and proteome results, we further analyzed the quantitative values of the relevant differential metabolites between AG and Hubbard fruits (Figure 5; Table S5). The results showed that glutathione content in AG fruits was higher than in Hubbard fruits, matching with the results of DEG and DEP analysis. Neoxanthin (a type of carotenoid) levels were also significantly increased in Hubbard fruits compared to AG fruits. Low levels of protoporphyrin (a chlorophyll biosynthesis precursor) and high levels of primary fluorescent chlorophyll catabolite were determined in Hubbard fruits, indicating decreased chlorophyll accumulation. It is speculated that the changes in carotenoid and chlorophyll accumulation contributed to the color deviation of fruits between these two pumpkin materials. Fruit enlargement is dependent on assimilate import from source leaves; therefore, larger fruits rely on more assimilate accumulation. In AG fruits, more D-glucose and stachyose were detected than in Hubbard fruits, whereas Hubbard fruits contained higher sucrose. The accumulation of raffinose did not show remarkable differences between AG and Hubbard fruits. Among the differential metabolites, we screened plant hormones that may be closely related to the regulation of fruit development and found that the content of JA was higher in large AG fruits, indicating that JA may play an important role in regulating the rapid expansion of AG fruits. The DEGs in the brassinolide biosynthesis pathway were identified in two types of pumpkin fruits, while brassinolides were not detected in the metabolome.

### 2.6. Transcription Factors Associated with Assimilate Accumulation

**Figure 6:**
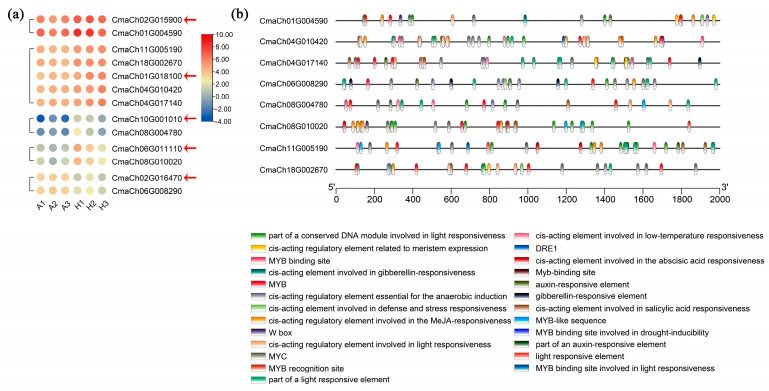
Identification of MYB transcription factors and assimilation metabolism-related genes that exhibited similar expression patterns. (**a**) Heatmap of selected MYB TFs and assimilation metabolism-related genes. The red arrows represent the MYB TFs with potential regulatory functions. (**b**) *Cis*-acting element analysis in the promoters of eight assimilation metabolism-related genes. A1~3 and H1~3 represent AG and Hubbard fruits, respectively.

The most typical characteristic of AG pumpkins was their significantly expanded fruits. Assimilate accumulation is one of the factors that promotes the expansion of pumpkin fruits. Hundreds of TFs were also differentially expressed between AG and Hubbard fruits. In order to preliminarily analyze the regulation mode of assimilate accumulation between AG and Hubbard fruits, a correlation assay between the differentially expressed TFs and the related genes involved in assimilate biosynthesis was evaluated. Several pairs of genes and MYB TFs with high expression correlation were found, including five MYB TFs and eight assimilation metabolism-related genes (Figure 6a; Table S6). *CmaCh02G015900*, *CmaCh01G018100*, *CmaCh10G001010*, *CmaCh06G011110*, and *CmaCh02G016470* were the identified MYB TF genes. *CmaCh04G010420* and *CmaCh04G017140* encode alpha-trehalose-phosphate synthase; *CmaCh11G005190* and *CmaCh08G010020* were annotated as sucrose-phosphate synthase genes; and *CmaCh08G004780* was a stachyose synthase gene. The above genes displayed lower expression in giant pumpkin fruits. Promoter analysis results indicated that the upstream promoter sequences of eight assimilation metabolism-related genes contained multiple MYB *cis*-acting elements, demonstrating that their expression may be regulated by MYB TFs (Figure 6b). Interestingly, in addition to *CmaCh08G004780*, the promoter region of seven other genes also contained *cis*-acting regulatory elements involved in MeJA responsiveness, suggesting that they also may be modulated by JA, which was associated with the metabolite’s variation.

**Figure 7:**
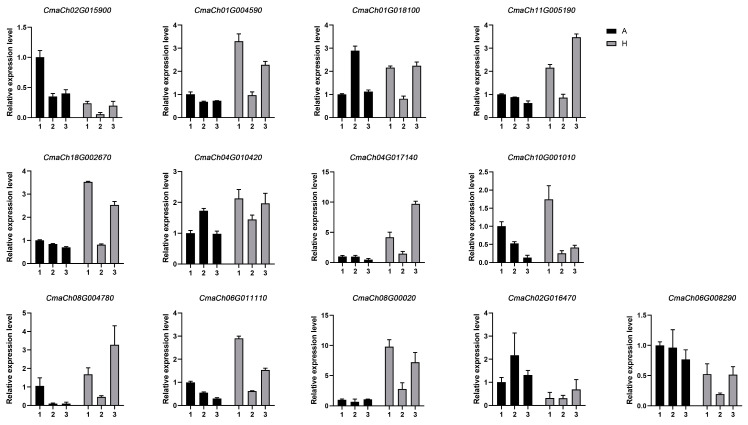
The expression levels of assimilation metabolism-related genes detected by the RT-qPCR assay. Data are expressed as the means ± standard deviation (SD) of three technical replicates.

In order to further confirm the potential regulatory relationship between the selected gene pairs, RT-qPCR analysis was carried out again to verify expression consistency. The results showed that three TF–gene pairs (*CmaCh02G015900* and *CmaCh01G004590* (encoding α-1,4 glucan phosphorylase), *CmaCh01G018100* and *CmaCh04G010420* (encoding α-trehalose-phosphate synthase), and *CmaCh06G011110* and *CmaCh08G010020* (encoding stachyose synthase)) shared high consistency in terms of gene expression (Figure 7). 

## 3. Discussion

Fruit size and color are important quality and yield traits of pumpkins [20]. The giant pumpkin (AG) produces the largest fruit in the world and holds great ornamental and economic value [21]. The AG cultivar is a modern cultivated pumpkin likely derived from its small-fruited ancestor (Hubbard), though its fruit present different colors and have expanded significantly in terms of size in contrast to the fruit of the Hubbard cultivar. However, the internal changes underlying the variation in fruit traits in giant pumpkin are not clearly defined. Herein, three omics approaches (transcriptome, proteome, and metabolome analysis) were employed to gain insights into crucial molecular and physiological processes related to the changes in color and size of giant pumpkin that developed from pumpkins with smaller fruit. 

The pigmentation of AG and Hubbard fruits respectively exhibit orange–yellow and orange–red coverage. Integrated multi-omics analysis demonstrated that the genes and proteins participating in photosynthesis showed high expression in Hubbard fruit, though this did not result in significantly increased fruit chlorophyll content, probably attributed to the fact that it was decomposed into primary fluorescent chlorophyll catabolite. In small-fruited pumpkin, high expression of chlorophyll synthesis-related genes did not correlate with accumulated chlorophyll. A possible explanation is that the growing period of AG and Hubbard pumpkins was inconsistent. At the rapid expansion stage, Hubbard fruit have just finished the process of fruit turning from green to orange, which is accompanied by chlorophyll degradation. Additionally, several lines of evidence have reported that carotenoids contribute to the regulation of fruit pigmentation [22,23]. In giant pumpkins, most of the DEGs related to carotenoid biosynthesis were down-regulated, and the detected neoxanthin level was significantly decreased in comparison to small pumpkins. Neoxanthin is a type of carotenoid that shows important functions in plants. Neoxanthin is also a pigment included in the LHC (light-harvesting chlorophyll-a/b) complex [24] and a precursor for abscisic acid (ABA) biosynthesis. When neoxanthin exists in the LHCII trimer, it can capture light and transfer energy to chlorophyll b [25]. When there is excess light energy, neoxanthin detaches from the LHCII trimer, which can quench singlet oxygen (^1^O_2_*) [25] and superoxide anions (O_2_^−^) [26], leading to the generation of abscisic acid [27]. Additionally, neoxanthin is involved in fruit pigmentation. It is reported that orange zucchinis obtain higher neoxanthin accumulation compared with light green and yellow zucchinis [22]. The color of pepper fruit also depends on the combined effects of anthocyanin, chlorophyll, and carotenoids [23]. In this study, the differences in the content of multiple carotenoids (including those with and without significant differences) may be responsible for the color difference exhibited by giant pumpkin. In mature pumpkin fruits, chlorophyll was degraded, with the difference in fruit color between AG and Hubbard fruits mainly caused by the change in carotenoid accumulation.

What makes a giant fruit? Researchers are currently paying more attention to this problem. A considerable number of studies indicate that the expansion of fruit is linked to assimilate accumulation and various plant hormones, including auxins, brassinosteroids, and cytokinins (CKs) [6,28,29]. Exogenous application of α-naphthaleneacetic acid (NAA) and 24-epibrassinolide (EBR) increased source capacity, transportation efficiency, and sink strength, resulting in the promotion of synthesis and the distribution of photoassimilates and ultimately increasing pumpkin fruit size [28]. In loquat, *BZR1* regulated cell expansion and fruit size by affecting brassinolide biosynthesis [30]. Maize *ZmBES1/BZR1-5* could control the expression of genes responsive to brassinosteroids and positively regulate kernel size [31]. Therefore, we speculated that the high expression of DEGs in the brassinolide synthesis pathway contributed to the formation of large fruit. Fruit size was determined by cell number and expansion. The faster fruit growth of the AG cultivar was driven by both faster cell proliferation and enlargement [6]. Overexpression of a CKs-inactivating enzyme gene (*AtCKX2*) in tomato fruit tissues resulted in reduced endogenous active CKs levels, a decreased number of cells, and smaller fruit size [29]. In a comparison between AG and Hubbard fruits, the identified differential metabolites did not contain auxins or cytokinins, while the accumulation of JA was significantly increased in giant pumpkin fruits. Similarly, a previous study reported that *SlHD8*, a downstream regulator of JA signaling, could promote tomato trichome elongation by directly binding to the promoters of a set of cell wall-loosening protein (expansins) genes and activating their expression [32]. It is well known that expansins regulate fruit size by controlling cell expansion [33,34]. According to transcriptome data, the expression levels of most DEGs that encode expansins were significantly up-regulated in giant pumpkins, speculating that the increase in JA accumulation might indirectly up-regulate the expression of expansin-encoded genes, thus leading to fruit expansion. 

The accumulation of photoassimilates was also one of the driving forces for the expansion of fruit or other product organs [35]. Numerous studies have reported that assimilate synthesis in leaves, the translocation of phloem, and partitioning into fruit are promoted in the giant pumpkin AG cultivar compared with small-fruited pumpkins [6]. In cucurbit crops, stachyose in RFOs was the main transport form of photoassimilates in vivo. In general, stachyose was synthesized from the source leaves and unloaded before being transported to the fruit (storage) through a long-distance transport and transformed into glucose as the source of substance and energy [36,37]. Thus, stachyose did not accumulate in the product organs [38]. In this study, the content of photoassimilates, including stachyose and D-glucose, was increased in giant pumpkin fruits, whereas the expression of the DEG-encoded stachyose synthase was lower in giant pumpkin fruit than in Hubbard fruit. We inferred that the transported stachyose had not been converted into glucose in time before it reached the giant pumpkin fruits owing to the rapid transportation. The detected stachyose in the fruit may come from the long-distance transportation of assimilates, rather than synthesis by the fruit itself. These findings provide evidence for the idea that stachyose has vital roles in giant pumpkin fruit development and present a new viewpoint on the unique unloading mode of assimilates in pumpkin. 

Photoassimilate accumulation relies on the transcription of genes involved in several metabolic pathways, including starch and sucrose metabolism. The expression of these genes was usually regulated by upstream TFs [39,40]. In order to further explore the regulation of photoassimilate metabolism, the TFs that showed higher expression consistency with DEGs in starch and sucrose metabolism were identified. Analysis of the TF–target genes pairs with consistent expression changes among different pumpkin samples, accompanied by prediction of *cis*-acting elements that existed in target gene promoters, revealed that several *MYB*s might play significant roles in regulating the transcription of DEGs in starch and sucrose metabolism. RT-qPCR results provided further evidence and highlighted the roles of *MYB*s in regulating the selected target genes in pumpkin, which were picked as the candidate upstream genes of these selected DEGs. Interestingly, the upstream promoter regions of eight selected DEGs in starch and sucrose metabolism also contained MeJA-responsive *cis*-acting elements, implying that gene expression may be regulated by JA accumulation [41]. It is speculated that exogenous spraying of JA or control of the expression of candidate MYB TFs might enhance the accumulation ability of assimilates in giant pumpkins, thus helping to further cultivate ornamental giant pumpkins with larger fruit.

Complex and dynamic networks of molecules are involved in plant fruit development. In the era of bioinformatics, huge amounts of multi-omics data have been generated and widely applied in botanical research [17,18,19]. However, a single layer of “omics” can only provide limited insights into the molecular regulatory mechanisms of crop traits. The speculated results generated from multi-omics assays still need to be further validated through a series of experiments (e.g., gene overexpression, gene knockout, and protein interaction validation). Moreover, fruit size is primarily determined by cell number, with cell size being the next significant determining factor. In our next project, changes in cell number during fruit formation will also be a focus of attention.

## 4. Materials and Methods

### 4.1. Plant Materials and Growth Conditions

The seeds of two *C. maxima* cultivars, AG (a cultivar with the world’s largest fruit) and Hubbard (a cultivar with smaller fruit), were purchased from the Sustainable Seed Company (Covelo, CA, USA). After seed germination, the seedlings were transferred at the three-leaf stage to a plastic greenhouse at Yangzhou University (119°260′ E, 32°240′ N). In the process of *C. maxima* cultivation, appropriate fertilizers and water management were provided according to the cultivation method of giant pumpkins [42]. The growth environment of the Hubbard and AG cultivars retained consistency. For each pumpkin plant, the second fruit at the main stem was allowed to grow while the other fruits were removed. The fruits of the two cultivars were then harvested at the rapid expansion stage (~30 nodes on the main stem). For the AG cultivar, the fruit was sampled at 30 days after flowering; for the Hubbard cultivar, the fruit was sampled at 20 days after flowering. The collected pumpkin samples were employed for transcriptome, proteome, and metabolome identification.

### 4.2. Transcriptome Analysis

Three biological samples were prepared for the transcriptome assay. Total RNA was extracted from AG and Hubbard fruits. A total amount of 1 μg of purified RNA with an RIN (RNA integrity number) > 7.0 per sample was used to construct cDNA libraries. The sequencing library was then prepared using an NEBNext UltraTM RNA Library Prep Kit (Illumina, San Diego, CA, USA) according to the manufacturer’s instructions. Subsequently, 100 bp of paired-end reads were generated by sequencing finished through the use of an Illumina platform. Library quality was assessed on the Agilent Bioanalyzer 2100 system. 

Raw data in fastq format were first processed through in-house perl scripts. In this step, clean data were obtained by removing the reads containing adapters, low-quality bases, and short sequences from raw data. Simultaneously, the Q20 values, Q30 values, GC content, and sequence duplication levels of the clean data were estimated. Generated clean reads with high quality were mapped to the *C. maxima* reference genome using Hisat (v2.2.1) software [43] and then used for all of the downstream assays. Functional annotation of obtained reference transcripts was achieved by referencing multiple databases, including the Nr (NCBI non-redundant protein sequences), Pfam (protein family), KOG/COG (clusters of orthologous groups of proteins), Swiss-Prot (a manually annotated and reviewed protein sequence database), KO (KEGG Ortholog database), and GO databases. The transcription levels of identified genes were normalized by calculating FPKM (fragments per kilobase million) values. The assay of DEGs between two sample groups was conducted using DESeq2 [44] based on the following criteria: |log2 FC| > 1.0 and false discovery rate (FDR) < 0.05. The functions of DEGs were clustered by metabolic pathways (KEGG) and GO terms.

### 4.3. Protein Extraction, Digestion, and LC-MS/MS

The fresh fruit samples from two pumpkin cultivars were prepared for proteomic experiments. Three biological replicates were set. Samples were dissolved in lysis buffer (7 M urea, 2 M thiourea, and 0.1% CHAPS). The plant tissue was then ground with three titanium dioxide abrasive beads (70 HZ, 120 s), followed by centrifugation at 5000× *g* for 5 min at 4 °C. Total protein was obtained from the collected supernatant. The concentration of the protein solution was measured using the Bradford protein assay. Subsequently, 200 μg of total protein from each sample was incubated with 5 μL of 200 mM dithiothreitol (DTT) at 55 °C for 1 h and then added to 5 μL of 375 mM iodoacetamide (held for 10 min at room temperature in darkness). Next, 200 μL of 100 mM dissolution buffer (AB Sciex, Framingham, MA, USA) was added and centrifuged. The sample peptides were digested in trypsin for 14 h and then freeze-dried and redissolved with 100 mM dissolution buffer for TMT labeling.

The peptides were suspended with 20 μL of buffer A (0.1% formic acid, 2% acetonitrile) and centrifuged at 12,000 rpm for 10 min. The supernatants (10 µL) were injected into a nano UPLC-MS/MS system. The samples were separated using an EASY-Spray C18 column. The mass spectrometer was operated in positive ion mode (source voltage 2.1 KV). Full MS scans were conducted in the Orbitrap with a scan range of 300–1500 m/z at a resolution of 120,000. For MS/MS scans, one full MS scan was followed by higher energy collisional dissociation fragmentation of the 20 most abundant ions with multiple charge states. The resultant peptide sequence data were matched against the Uniprot_HUMAN database in this experiment. Obtained MS/MS data were processed using Proteome Discoverer 2.4 from Thermo Fisher Scientific (Waltham, MA, USA). 

Protein identification was based on the following settings: precursor ion mass tolerance, ±15 ppm; fragment ion mass tolerance, ±0.5 Da; maximum missed cleavages, 2; static modification, carbox yamidomethylation (57.021 Da) of Cys residues; dynamic modifications, oxidation modification (+15.995 Da) of Met residues. The *p* value of primary data was calculated, and data with a *p* value ≤ 0.05 and a difference ratio ≥ 1.2 were selected for further analysis.

### 4.4. Metabolites Identification

Six biological replicates of AG and Hubbard fruits were sampled for non-targeted metabolite profiling. About 50 mg of the ground pumpkin sample was mixed with 1 mL of extract (methanol:acetonitrile:water, 2:2:1, *v*/*v*) containing an internal standard (20 mg/L) and then vortexed. Ultrasound extraction in an ice water bath was applied to the resulting mixture for 10 min, followed by stewing for 1 h at −20 °C. Subsequently, the samples were centrifuged at 12,000 rpm for 15 min at 4 °C, and the supernatant hosting metabolites was obtained. The collected supernatant was dried in a speed vacuum and 160 μL of extract (acetonitrile:water, 1:1, *v*/*v*) was then added. The mixture was vortexed and used for ultrasound extraction before then being centrifuged at low temperature. 

A total of 120 μL of supernatant was loaded into the LC-QTOF MS system (Acquity I-Class PLUS ultra-high-performance liquid tandem Waters Xevo G2-XS QTOF high-resolution mass spectrometer; Waters, Manchester, UK) [45]. The liquid chromatograph column was a Waters Acquity UPLC HSS T3 column (1.8 μm 2.1 × 100 mm). Metabolite determination was achieved through the use of two modes (positive and negative ions). The parameters of the ESI ion source were set as follows: capillary voltage, 2000 V (positive ion mode) or −1500 V (negative ion mode); cone voltage, 30 V; ion source temperature, 150 °C; desolvent gas temperature, 500 °C; backflush gas flow rate, 50 L/h; desolventizing gas flow rate, 800 L/h. 

Based on the untargeted LC-QTOF MS runs, the data matrix was aligned using MassLynx (v 4.2) software (Waters, Milford, MA, USA). The raw data preprocessed by MassLynx (v4.2) were further processed by Progenesis QI (v2.0) software for peak extraction, peak alignment, and other data processing operations according to the Progenesis QI software online METLIN database and Biomark’s self-built library for identification. Both theoretical fragment identification and mass deviation were within 100 ppm. After normalizing the original peak area information with the total peak area, follow-up analysis was performed. PCA and Spearman correlation analysis were adopted to evaluate the repeatability of the samples within the group. Identified metabolites were searched for classification and pathway information in the KEGG, HMDB, and LIPID MAPS databases. Following the grouping information, the difference multiples were calculated, and the significance of the difference (*p* value) of each metabolite was analyzed using the *t*-test. The OPLS-DA model was constructed using the R language package ropls, and the reliability of this model was verified through 200 times permutation tests. Multiple cross-validation was employed to calculate the VIP value of the model. Differentially expressed metabolites were screened using the method of combining the difference multiple, the *p* value, and the VIP value with a selection criterion (FC > *p* value < 0.05 and VIP >1). The significantly enriched KEGG pathway of differential metabolites was calculated using a hypergeometric distribution test. Metabolome data statistics were obtained using the BMKCloud platform (Biomarker Technologies Corporation, Beijing, China).

### 4.5. Assay of Cis-Acting Elements in Promoters

To investigate the candidate regulatory factor of genes in starch and sucrose metabolism, we retrieved the 2000 bp long sequences in the target gene promotor from the *C. maxima* genome database [46]. The known *cis*-elements in the selected target gene promotor were surveyed through searches of the PlantCARE database [47]. 

### 4.6. Real-Time Quantitative PCR 

To further validate the expression consistency of selected MYB and photoassimilate metabolism-related genes, an RT-qPCR assay was performed using the CFX-96 system (Bio-Rad, Hercules, CA, USA) and the iTaq Universal SYBR^®^ Green Supermix Kit (Bio-Rad, Hercules, CA, USA). Primer Premier 6.0 software was employed to design the RT-qPCR primers of target genes, and the sequences are listed in Table S7. To provide higher reference values for co-expression results, the pumpkin samples used for RT-qPCR detection differed from those used for transcriptome sequencing. Data were analyzed using Microsoft Office Excel (2019), and the relative expression level of detected genes was calculated using the 2^−ΔΔCt^ method [48]. *EF-1α* was applied as the internal reference for data normalization [49].

## 5. Conclusions

**Figure 8:**
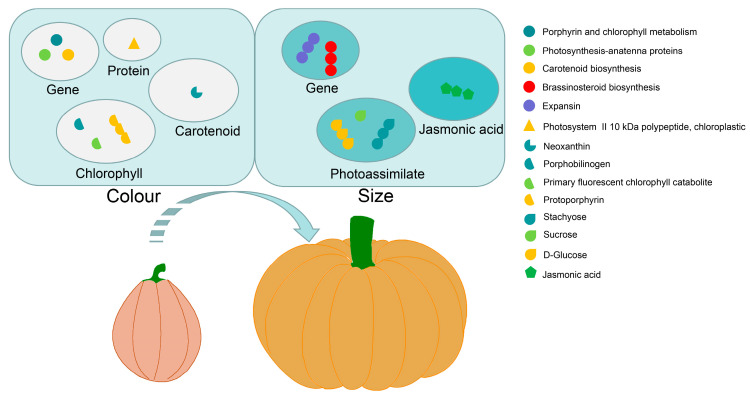
A comprehensive scheme summarizing changes in the transcription of various genes, protein expression, and metabolites from small-fruited pumpkin to giant pumpkin. Single marks and three interlinked marks represent down-regulation and up-regulation, respectively.

In summary, our findings provide a comprehensive view of the gene, protein expression, and metabolite diversity observed in the fruits of giant pumpkins and small-fruited pumpkins (Figure 8). The differences in chlorophyll and carotenoid content altered the pigmentation of giant pumpkin fruit. The up-regulation of DEG-encoded expansins contributed to fruit cell expansion, while increased stachyose accumulation and JA regulation worked together for the formation of large fruit in giant pumpkin. The current investigation reveals the potential formation factors of giant pumpkins and improves our knowledge concerning variations in fruit color and size. The findings in this study also provide a great reference for cultivating new pumpkin resources with different fruit size and color.

## Data Availability

The data supporting the conclusions of this article are listed in the text and Supplementary Materials.

## Supplementary Materials

The following supporting information can be downloaded at: https://www.mdpi.com/article/10.3390/ijms25073864/s1.
