# Supplementary material for: Multi-Omics Analysis Reveals the Distinct Features of Metabolism Pathways Supporting the Fruit Size and Color Variation of Giant Pumpkin
Source: Int J Mol Sci. 2024 Mar 29;25(7):3864. doi: 10.3390/ijms25073864 (PMC11012166; doi:10.3390/ijms25073864)
Supplement: Supplementary file 1 [file ijms-25-03864-s001.zip › Supplementary Figures-0229.pdf]

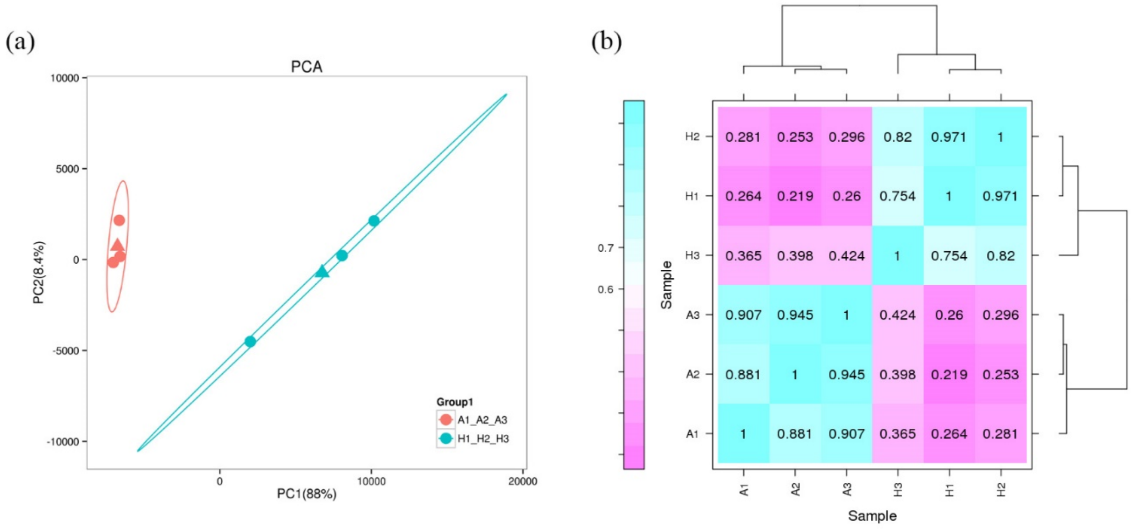

Figure S1 Inter-sample correlation analysis based on transcriptomics data. (**a**) Principal component plot of the individual samples based on count matrix. (**b**) A sample hierarchical clustering based on the heatmap of sample-sample distances. Distance heatmap computed from the count matrix.

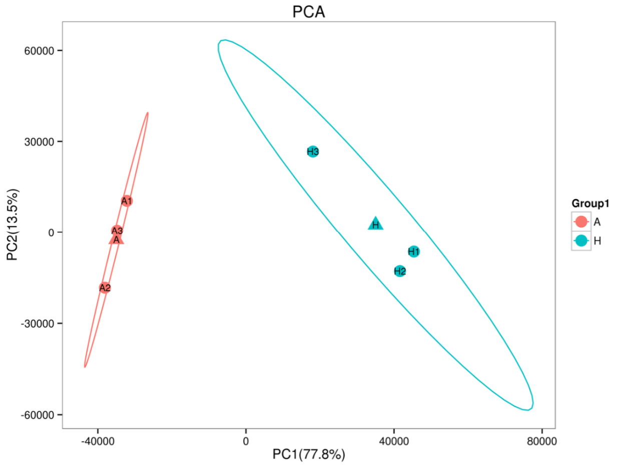

Figure S2 Principal component analysis of the individual samples used for proteome analysis.

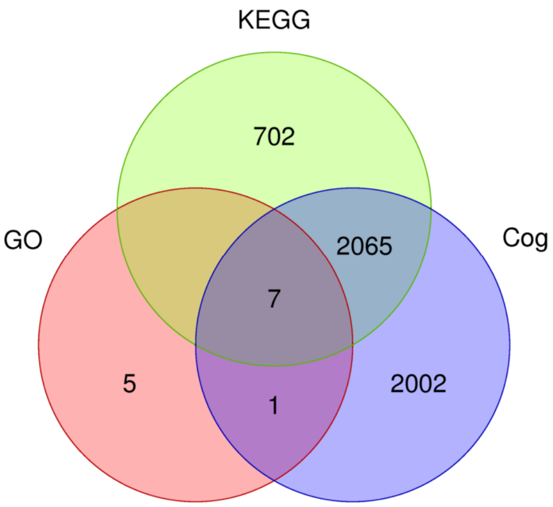

Figure S3 Venn diagram of protein function annotation results based on proteomics data.

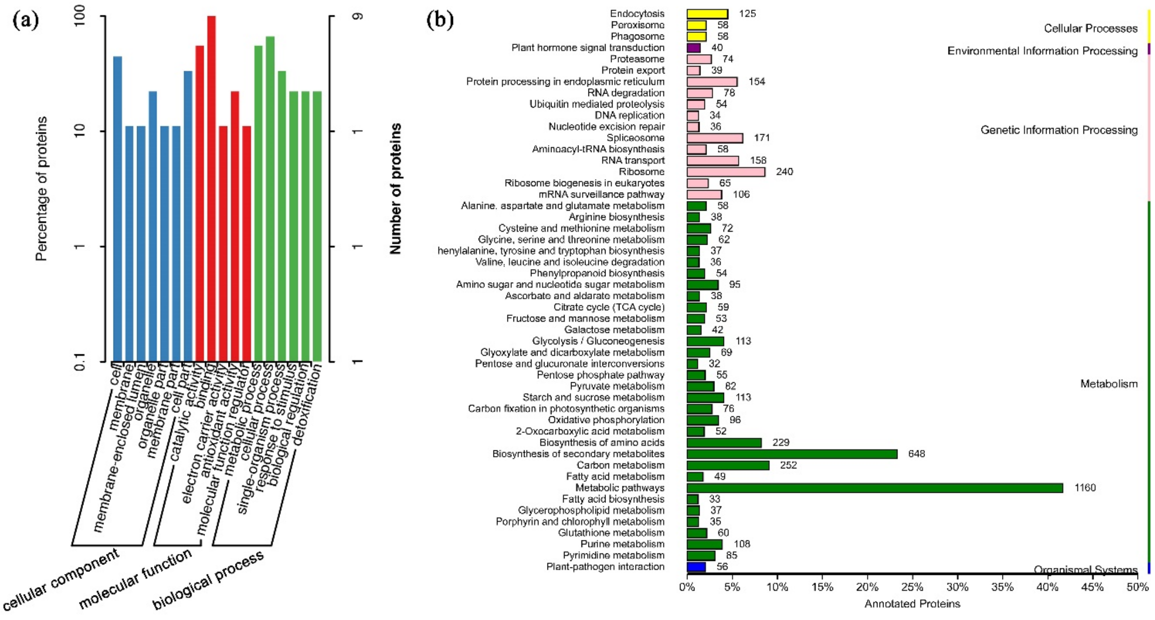

Figure S4 Annotation and pathway analysis of the identified proteins from proteome data.

(**a**) GO classification. (**b**) KEGG pathway.

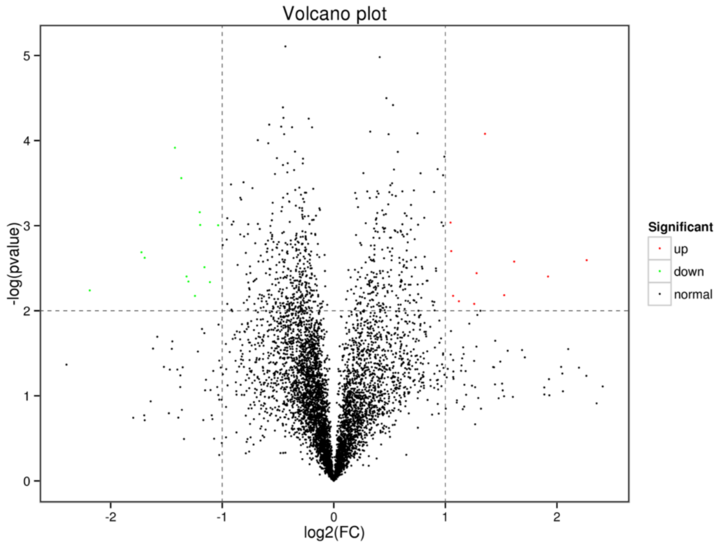

Figure S5 Volcano plot of differentially expressed proteins (DEPs).

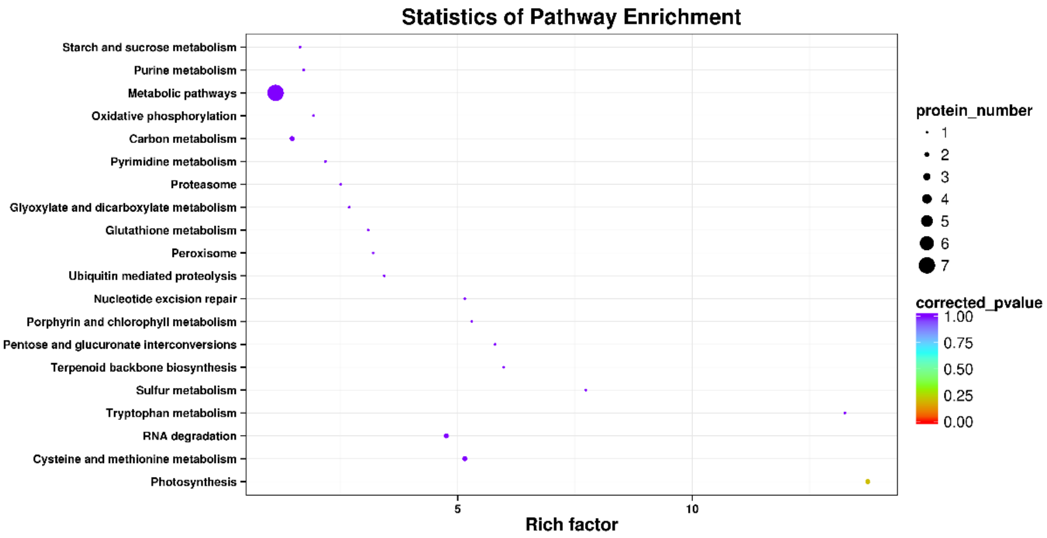

Figure S6 Scatter plot of the most enriched KEGG pathways of all DEPs.
